# Prey-Mediated Effects of Drought on the Consumption Rates of Coccinellid Predators of *Elatobium abietinum*

**DOI:** 10.3390/insects7040049

**Published:** 2016-09-27

**Authors:** Jennifer A. Banfield-Zanin, Simon R. Leather

**Affiliations:** 1 Department of Life Sciences, Silwood Park, Imperial College London, Ascot SL5 7PY, UK; 2 Centre for Integrated Pest Management, Harper Adams University, Newport TF10 8NB, UK; sleather@harper-adams.ac.uk

**Keywords:** *Elatobium abietinum*, *Picea sitchensis*, *Aphidecta obliterata*, *Adalia bipunctata*, drought stress, consumption rate, climate change, biological control

## Abstract

Climate change in the UK is predicted to cause an increase in summer drought events. *Elatobium abietinum* is an important pest of Sitka spruce (*Picea sitchensis*), causing defoliation of trees, and is predicted to become more abundant in response to climatic change, reducing spruce productivity. Populations are also moderated by invertebrate predators, though the extent to which this might be modified under a changing climate is unclear. *Elatobium abietinum* is preyed upon by the coccinellid species *Aphidecta obliterata* (a spruce specialist) and *Adalia bipunctata* (a generalist), populations of which naturally occur in spruce plantations. This study sought to investigate the effect of different intensities and frequencies of drought on the consumption rate of the aphids by the two coccinellids. In Petri dish trials, severe drought stress increased the consumption rates of 3rd instar aphids by both adult and larval coccinellids. Moderate intermittent stress tended to result in a reduced consumption rate for larval coccinellids only, suggesting an age-dependent response. The findings of this study suggest that, under drought conditions, a prey-mediated effect on predator consumption, and, therefore, biocontrol efficacy, is likely, with drought intensity and frequency playing an important role in determining the nature of the response.

## 1. Introduction

There is an increasing body of evidence supporting claims that climate change will alter presence, distribution, abundance, physiology and population dynamics of plants, their insect herbivores and the natural enemies of those herbivores [1,2,3]. Underpinning such changes are the effects of climate alteration on various life history parameters, including development times, survival and reproductive rates. Temperature, for example, has a direct impact on insects, with studies showing that simulated climate warming may increase development rates and voltinism [4], additionally altering feeding rates [1]. Changes to plant physiology may be expected under drought stress, which, in turn, also alter insect herbivore performance and behaviour [5,6,7], then indirectly affecting natural enemy performance and abundance [8]. Natural enemy performance and behaviour has also been shown to be more directly affected by drought [9], altering top-down effects and foodweb dynamics [10], and predator diversity [11].

Clear, generalised predictions in relation to the response of plant-herbivore interactions under water deficit are difficult to draw. Drought stress has been observed to have either detrimental or beneficial effects on herbivore performance, survival and population dynamics [4,12,13,14,15,16]. Such variations are attributed to the magnitude and frequency of the drought event [6,7], which, while enhancing host nutritional quality, may also render, in the case of aphids, the phloem sap inaccessible due to reduced turgor pressure depending on the intensity and duration of the water deficit bout. Such uncertainty is also compounded by differences in plant [17,18] and herbivore [5] species-specific responses, and the dependence of such responses to other abiotic factors including temperature [8].

Less evidence is available on the predicted effects of climate change on natural enemies. A disruption to multi-trophic level interactions may well be expected in response, driven by differing responses between community members and trophic levels [2,19,20]. This could potentially lead to altered food web and ecosystem stability [21]. Increasing temperature, for example, may lead to altered insect phenologies, and temporal and spatial mismatches between herbivores and their natural enemies [22]. Additionally, natural enemies are often more sensitive to local extinctions following environmental changes when compared to other trophic levels [22,23]. Prey-mediated effects may also complicate the response. Host-induced changes to insect herbivore quality, often through changes to plant secondary metabolites, have been shown to mediate the tritrophic interaction [24,25,26], with changes to consumption rates and functional responses also observed in response to host plant quality [27,28,29]. Furthermore, by altering insect herbivore performance and survival, drought stress can result in changes to prey population demography, which can, in turn, alter predation/parasitisation rates due to prey/host suitability [30,31].

Natural enemies are believed to play an important role in driving certain aspects of the population dynamics exhibited by green spruce aphids, *Elatobium abietinum* (Walker) (Hemiptera: Aphididae) [32], a major defoliating pest of Sitka spruce (*Picea sitchensis* (Bong.) (Carr.)) in the United Kingdom. The cyclical dynamics result not only from climate and density-dependant processes, which affect both overwinter survival and consequent spring peak size, but also a delayed density-dependent impact of predators and parasitoids [33]. The latter is thought to help maintain low aphid population size in the years following a major outbreak [34], as well as contributing to driving the population decline following the spring peak [32,35]. A variety of natural enemy families and species have been found to be associated with *E. abietinum* including, but not limited to, Coccinellidae (and several other Coleopteran families), Hemerobiidae (Neuroptera), Syrphidae (Diptera), and various Hymenopteran parasitoids (including Aphididae and Aphelinidiae) [36,37]. Among the various groups of natural enemies, coccinellids were the most abundant, and of the coccinellid species present, the larch ladybird, *Aphidecta obliterata* (L.), was predominant [37,38].

Climate change predictions for the UK stipulate warmer, milder winters and increased frequency of summer drought events in hot, dry summers [39,40]. As a result of these, *E. abietinum* is expected to increase in pest status [35]. Sitka spruce was introduced to Great Britain from North America [41] and is dependent on the presence of abundant moisture during the growing season. It has been planted extensively in regions with a maritime climate (characterised by mild winters and wet, relatively cool summers) [42,43,44], and is intolerant of drought [45]. It is therefore important to understand the potential nature of the interactions between this pest species and its natural enemies. Coccinellids are important predators of aphids [46], and are the most abundant predators of *E. abietinum* on spruce [37,47]. In order to understand their potential in the control of *E. abietinum* under future climate conditions or to make predictions on their interaction, understanding the potential tritrophic effects of drought are essential. *Aphidecta obliterata* is a spruce specialist, and *Adalia bipunctata* (L.), though not always associated with *E. abietinum*, is an arboreal generalist and was found by Leather and Owuor [47] to be the most abundant predator on Norway spruce (*Picea abies* L.) at Silwood Park (Ascot, UK).

The present study sought to investigate the effect of spring-summer drought stress on the 24 h consumption rates of specialist and generalist coccinellid predators of *E. abietinum* as adults and larvae, and to experimentally determine whether any effects were prey- or plant-mediated. It tested the hypothesis that moderate intermittent drought stress levels, previously found to be beneficial for *E. abietinum* growth and size [14], would reduce consumption rates, while severe levels of water deficit, detrimental to *E. abietinum* growth and size [14], would lead to increased consumption rates. This would have implications for future predation pressure and control levels in Sitka spruce plantations, influencing damage levels on the conifer crop under changing climate.

## 2. Materials and Methods

### 2.1. Drought Treatments

Five drought levels were simulated in two-year-old potted Sitka spruce saplings: FC—plants maintained at field capacity (control); MS—plants maintained at 60% field capacity; CS—plants maintained at 20% field capacity; MIS—plants subjected to intermittent stress, where pots were allowed to fluctuate between 30% and 70% field capacity; SIS—pots were allowed to fluctuate between 20% of field capacity and field capacity (where field capacity was defined as the weight of saturated growing medium after one hour of free drainage). A combination of soil volumetric water content (determined with an SM200 soil moisture sensor and an HH2 meter; Delta-T Devices, Cambridge, UK), and pot weight was used to monitor soil moisture levels.

### 2.2. Plant Material

#### 2.2.1. Material for Petri Dish Arena Cuttings

Fifty two-year-old Sitka spruce saplings (vegetatively propagated, Ident. QSS 04 (0R18TE)) were potted using a standard 2:1:1 peat, bark and perlite growing medium in 3 L pots. A controlled release granular fertiliser (20 g Osmacote^®^ Plus; 16% N + 8% P + 11% K + 2% MgO; Scotts Ltd., UK) was also added to the potting mixture. Saplings were randomly assigned to one of the five drought treatments, such that there were ten saplings per treatment. Pots were placed outdoors on raised pallets, and drought treatments maintained from the beginning of March to the end of October. They were watered using an automated irrigation system, which was monitored daily to ensure the correct moisture content of the soil. A sealed plastic skirt was applied to the base of each tree, in order to exclude rainwater. At the end of the drought treatment, the plastic skirts were removed and the saplings allowed to overwinter. The following February, the now three-year-old saplings were re-potted into 7 L pots and fully watered for three weeks to allow establishment. The plastic skirts were then reattached and drought treatments started again. Throughout the entire preparation period, plants were checked daily for aphids and any that were found were removed.

#### 2.2.2. Material for *E. abietinum* Cultures

Fifty two-year-old Sitka spruce saplings (vegetatively propagated, Ident. QSS 04 (0R18TE)) were obtained and potted up as per the arena cutting Sitka spruce in the second year of the experiment. They were kept in a greenhouse under a minimum of $20{\;}^{\circ }C$, ambient humidity and 16L:8D photoperiod. After potting, these were fully watered to allow establishment for three weeks before drought treatment was applied. After two months, the plants were moved outdoors under continued drought treatment and under the same maintenance as the arena Sitka spruce saplings. After an additional two months, these were then moved to the rearing room under continued drought treatment and left to acclimatise for two weeks before aphid inoculation.

### 2.3. Insect Cultures

#### 2.3.1. Aphids

Cultures of *E. abietinum* were reared on fifty-two-year-old Sitka spruce saplings, with ten saplings per drought treatment, at $15{\;}^{\circ }C$, 70% relative humidity (RH) and 16L:8D photoperiod. The saplings were artificially inoculated with cuttings taken from a stock culture maintained on cut branches of Sitka spruce, obtained from Alice Holt Forest Research Station (Surrey, UK), in buckets of water.

A second aphid, *Rhophalosiphum padi* (L.), used for feeding coccinellid cultures was reared on potted barley (*Hordeum vulgare* (L.)) at $15{\;}^{\circ }C$, 70% RH and 16L:8D and kept in insect cages.

#### 2.3.2. Coccinellids

Cultures of *A. obliterata* and *A. bipunctata* were maintained separately from the aphids at $15{\;}^{\circ }C$, 70% RH and 16L:8D. They were reared in 14 cm × 9.5 cm× 26.5 cm perspex boxes, each of which had two large muslin-covered holes in the lid. A folded filter paper was provided in each box as a suitable egg-laying surface. Boxes were examined daily and any eggs removed and placed in separate perspex boxes until hatching. Both adult and larval coccinellids were fed daily ad libitum on *R. padi*, a suitable substitute for *E. abietinum* [28,37].

### 2.4. Consumption in a Petri Dish

The consumption rate experiments were all conducted in the coccinellid culture controlled temperature (CT) room, which was kept at $15{\;}^{\circ }C$, with 70% RH and a 16L:8D photoperiod. Aphid numbers offered were selected to represent ad libitum availability, as per Timms [28,37]. Third instar aphids were utilised as prey items, as these would not produce additional young during the experiment’s duration.

#### 2.4.1. Adult Consumption

Seventy-five adult *A. obliterata* and *A. bipunctata* were placed into individual 9 cm Petri dishes and starved for 24 h. They were then transferred into a new 9 cm Petri dish with Fluon^®^-coated sides (this was to prevent aphids from walking onto the top of the Petri dish). Each of these new Petri dishes contained 100 approximately 3rd instar *E. abietinum* aphids, sourced from the culture trees for each drought treatment. The coccinellids were then left for a further 24 h to feed before being removed, and the number of aphids consumed in that period of time recorded.

#### 2.4.2. Larval Consumption

One-hundred-and-fifty 1st instar *A. obliterata* and *A. bipunctata* larvae were placed into individual 9 cm Petri dishes with Fluon^®^-coated sides within 12 h of hatching. Each Petri dish contained fifty approximately 3rd instar *E. abietinum* aphids, sourced from the culture trees for each drought treatment. The larvae were then left for 24 h to feed before being removed, and the number of aphids consumed in that period of time recorded.

### 2.5. Consumption on Host Plant Material

The above methodology was repeated, for both adults and larvae of each coccinellid species, in the presence of Sitka spruce plant material.

A 4 cm segment of Sitka spruce side-branch, sourced from the three-year-old Sitka spruce left outdoors, was placed into each Petri dish after having been carefully examined for aphids (which were removed from the segments). The appropriate number of 3rd instar *E. abietinum* aphids were then added, and an inverted Petri dish base was attached securely to the top to allow enough space for the spruce needles. The sides of all Petri dish bases were coated with Fluon^®^. After two hours, any aphids which had not moved onto the spruce needles were moved there using a fine paintbrush and left for a further hour. A coccinellid was then placed onto each spruce segment and left for 24 h, after which they were removed and the number of aphids consumed recorded. Each needle was carefully removed from the segment stem before the remaining section of stem was carefully examined for aphids, to make sure no aphids were missed.

### 2.6. Statistical Analysis

All statistical analyses were carried out using the statistical program, R (version 2.11.0; [48]). The effects of drought stress and host plant material presence were assessed using linear mixed effect models, using the ‘lme4’ package [49] as per Bolker et al. [50], and checked for significance using the ‘car’ package [51]. Drought was modelled as a fixed effect, while coccinellid weight and the tree from which prey aphids were obtained were modelled as random effects (for each species of adult coccinellid groups: tree = 5, weight = 75; *n* = 5, estimated d.f. for each parameter = 4. For each species of coccinellid larvae groups: tree = 10, weight = 150; *n* = 10, estimated d.f. for each parameter = 4). A post hoc Tukey’s HSD test was used to compare between drought treatments where significance was observed, using the ‘multcomp’ package [52]. Model simplification was carried out and tested with ANOVA where appropriate, as per Crawley [53].

## 3. Results

### 3.1. Effects on Adult Coccinellids

#### 3.1.1. *Aphidecta obliterata* Adults

An effect of both drought ($\chi^{2}$${}_{4}$ = 94.11, *p* < 0.001) and host plant presence/absence ($\chi^{2}$${}_{1}$ = 14.64, *p* < 0.001) on aphid consumption were found through model simplification, although no interaction was observed (*p* > 0.05). A smaller number of aphids was consumed across the treatments when a segment of Sitka spruce was included in the Petri dish (Table 1).

**Figure 1:**
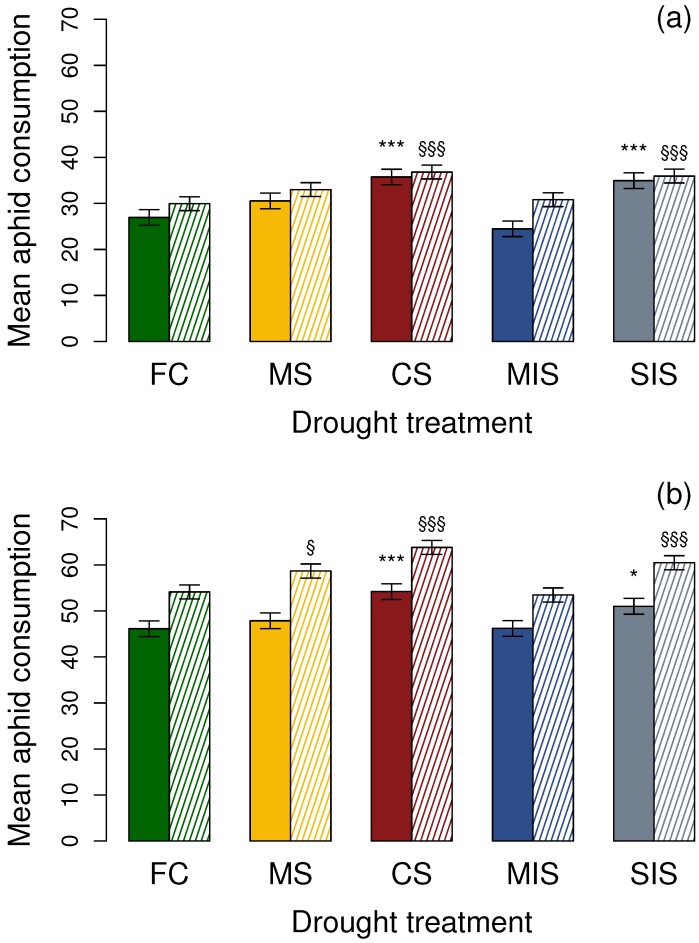
The effect of drought stress on mean number of 3rd instar *E. abietinum* consumption by (**a**) *Aphidecta obliterata* and (**b**) *Adalia bipunctata* adults, as number consumed over 24 h ± LSD. Solid colours = in presence of a Sitka spruce segment; Dashed = no Sitka spruce segment. Error bars and significance indicated against FC with respect to host plant presence or absence. FC = field capacity; MS = 60% field capacity; CS = 20% field capacity; MIS = allowed to fluctuate from 70% to 30% field capacity; SIS = allowed to fluctuate from field capacity to 20% field capacity. *** or §§§ = *p* < 0.001; ** or §§ = *p* < 0.01; * or § = *p* < 0.05.

When a Sitka spruce segment was included in the Petri dish, the consumption rate of aphids by *A. obliterata* adults was affected by drought stress level ($\chi^{2}$${}_{4}$ = 66.95, *p* < 0.001; Figure 1a), where a greater number of aphids were consumed under the severe level drought treatments, CS (*z* = 5.19, *p* < 0.001) and SIS (*z* = 4.72, *p* < 0.001). The consumption rates were not found to differ between the FC control and moderate drought stress treatments, MS (*z* = 2.12, *p* > 0.05) and MIS (*z* = −1.45, *p* > 0.05).

In a Petri dish with no Sitka spruce segment, a similar pattern was observed. Drought was found to affect the number of aphids consumed by *A. obliterata* adults ($\chi^{2}$${}_{4}$ = 28.80, *p* < 0.001; Figure 1a), where a greater number of aphids were consumed under both of the severe level treatments, CS (*z* = 4.29, *p* < 0.001) and SIS (*z* = 3.75, *p* < 0.001). No significant difference was observed between the consumption rates under the FC control and that of the moderate drought level treatments (MS, *z* = 1.92, *p* > 0.05; MIS, *z* = 0.54, *p* > 0.05).

#### 3.1.2. *Adalia bipunctata* Adults

Similarly to *A. obliterata*, a significant effect of both drought ($\chi^{2}$${}_{4}$ = 69.07, *p* < 0.001) and host plant presence/absence, where more aphids were consumed in an empty Petri dish ($\chi^{2}$${}_{1}$ = 163.29, *p* < 0.001), were found through model simplification for *A. bipunctata* adults (Table 1). No interactions between the two variables were found.

In the presence of a Sitka spruce segment in the Petri dish, the consumption rate of aphids by *A. bipunctata* adults was affected by drought stress level ($\chi^{2}$${}_{4}$ = 32.79, *p* < 0.001; Figure 1b), where a greater number of aphids were consumed under the severe level drought treatments, CS (*z* = 4.70, *p* < 0.001) and SIS (*z* = 2.83, *p* < 0.05). The consumption rates were not found to differ between the FC control and moderate drought level treatments (MS, *z* = 1.01, *p* > 0.05; MIS, *z* = 0.04, *p* > 0.05).

In the absence of a Sitka spruce segment, adult *A. bipunctata* consumption of *E. abietinum* was also found to be affected by drought ($\chi^{2}$${}_{4}$ = 58.94, *p* < 0.001; Figure 1b), where a significantly greater number of aphids were consumed under the MS, CS and SIS drought treatments (MS, *z* = 2.83, *p* < 0.05; CS, *z* = 6.04, *p* < 0.001; SIS, *z* = 3.95, *p* < 0.001). Consumption of aphids reared under the MIS drought treatment showed no significant difference to that under the FC control (*z* = −0.42, *p* > 0.05).

### 3.2. Effects on 1st Instar Coccinellid Larvae

#### 3.2.1. *Aphidecta obliterata* Larvae

Both drought ($\chi^{2}$${}_{4}$ = 146.60, *p* < 0.001) and host plant presence/absence ($\chi^{2}$${}_{1}$ = 11.76, *p* < 0.001) were shown to have a significant effect on the number of aphids consumed by 1st instar larvae of *A. obliterata* over 24 h. Fewer aphids, however, rather than more, were consumed in an empty Petri dish by the larvae (Table 2). No interactions were observed between drought and host plant presence/absence.

**Figure 2:**
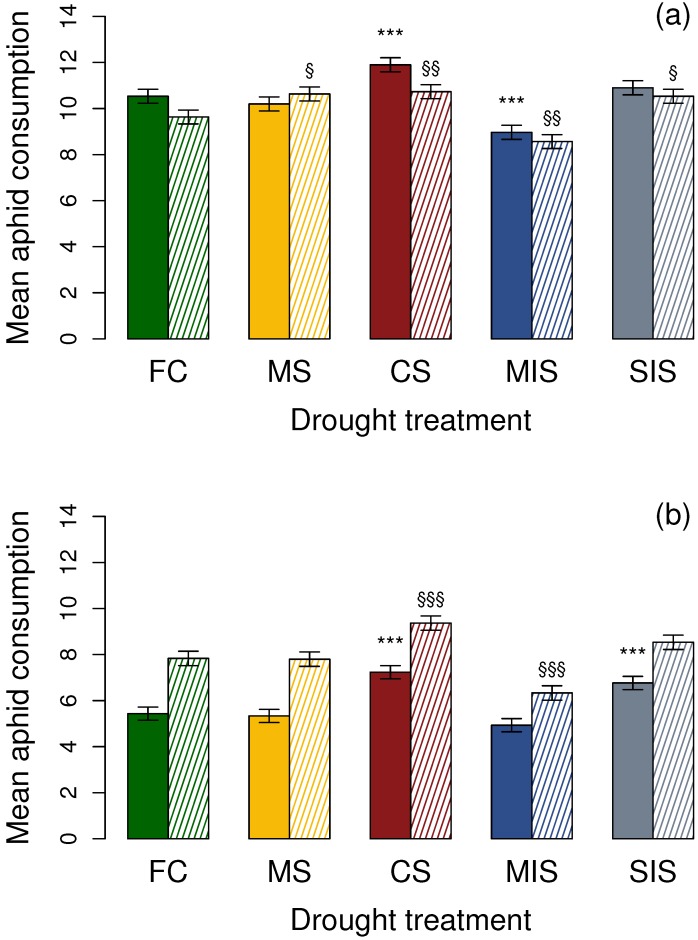
The effect of drought stress on mean number of 3rd instar *E. abietinum* consumption by (**a**) *Aphidecta obliterata* and (**b**) *Adalia bipunctata* 1st instar larvae, as number consumed over 24 h ± LSD. Solid colours = in presence of a Sitka spruce segment; Dashed = no Sitka spruce segment. Error bars and significance indicated against FC with respect to host plant presence or absence. FC = field capacity; MS = 60% field capacity; CS = 20% field capacity; MIS = allowed to fluctuate from 70% to 30% field capacity; SIS = allowed to fluctuate from field capacity to 20% field capacity. *** or §§§ = *p* < 0.001; ** or §§ = *p* < 0.01; * or § = *p* < 0.05.

*Aphidecta obliterata* larvae in a Petri dish, which included a Sitka spruce segment ($\chi^{2}$${}_{4}$ = 67.96, *p* < 0.001, Figure 2a), were found to consume a greater number of aphids reared under CS drought treatment (*z* = 3.73, *p* < 0.001), while consuming significantly fewer aphids reared under the MIS drought treatment (*z* = −4.28, *p* < 0.001). There was no significant difference between the number of aphids eaten by the larvae in both the MS and SIS drought treatments (MS, *z* = −0.91, *p* > 0.05; SIS, *z* = 1.00, *p* > 0.05) and the larvae in the FC control drought treatment.

In the absence of host plant material, consumption rates under all drought treatments showed a significant difference from the FC control ($\chi^{2}$${}_{4}$ = 60.66, *p* < 0.001; Figure 2a). More aphids were consumed under the MS, CS and SIS treatments (MS, *z* = 2.74, *p* < 0.05; CS, *z* = 3.27, *p* < 0.01; SIS, *z* = 2.61, *p* < 0.05), while fewer were eaten under the MIS drought treatment (*z* = 3.33, *p* < 0.01).

#### 3.2.2. *Adalia bipunctata* Larvae

*Adalia bipunctata* larvae showed a significant response under both drought ($\chi^{2}$${}_{4}$ = 185.34, *p* < 0.001) and host plant presence/absence ($\chi^{2}$${}_{1}$ = 221.44, *p* < 0.001) (Table 1). No interaction between the two variables was observed.

When presented with aphids on a Sitka spruce segment, the consumption of *E. abietinum* aphids by *A. bipunctata* larvae was affected by drought level ($\chi^{2}$${}_{4}$ = 95.63, *p* < 0.001; Figure 2b). A greater number of aphids reared under the severe drought treatments, CS and SIS, were consumed (CS, z = 6.26, *p* < 0.001; SIS, z = 4.65, *p* < 0.001). There was no effect on consumption under the moderate MS and MIS drought treatments (MS, z = −0.20, *p* > 0.05; MIS, z = −1.72, *p* > 0.05).

In the absence of a Sitka spruce segment ($\chi^{2}$${}_{4}$ = 100.55, *p* < 0.001; Figure 2b), there was no difference in the number of aphids consumed under the MS and SIS treatments (MS, *z* = 0.11, *p* > 0.05; SIS, *z* = 2.22, *p* > 0.05) when compared to consumption rates under the FC control. Furthermore, while more aphids were consumed under the severe CS drought treatment (*z* = 4.87, *p* < 0.001), significantly fewer were consumed under the MIS drought treatment (*z* = −4.76, *p* < 0.001).

## 4. Discussion

Changes to abiotic conditions associated with climate change, such as increased temperature and atmospheric gases, have repeatedly been shown to affect phytophagous insects [54]. The same is true for drought stress [4,12,13,14,15,16]. The Pulsed Water Stress hypothesis [6] proposed that intermittent stress would benefit phloem-feeding insects. The hypothesis was further refined by Mody et al. [7] who highlighted the importance of stress magnitude. Despite this, a meta-analysis by Koricheva et al. [12] indicated that drought reduced the reproductive potential of sucking insects.

The effects of drought on plants themselves range from effects at a cellular level [55] and on plant chemistry [56,57,58,59], through to changes to plant structure [55,60] and growth [60,61,62]. Furthermore, by modifying the nutrient uptake by a plant [63], drought also has the potential to affect phloem sap quality for phloem-feeding insects such as aphids, much as turgor pressure affects availability of the sap to the herbivores.

In order to understand the predatory performance of natural enemies under drought stress, it is necessary to first understand the effects on their phytophagous prey. Water stress has been shown to affect the host preference of insects. The wood borer *Tomicus destruens* (Coleoptera: Scolytidae) was found to not only have a preference for well-watered pine, but also to have higher survival rates on those plants when compared to water stressed plants [57]. Another borer, *Hylotropus bajulus* (Coleoptera: Cerambycidae), however, showed no difference in performance between well-watered and stressed pine seedlings [55]. On the other hand, leaf-cutting *Atta* ants (Hymenoptera: Formicidae) had a preference for drought stressed plants [64]. By affecting host preference, and given that drought rarely has an even effect across a tree stand [65], changes to prey distribution could be observed, which, in turn, may affect accessibility to natural enemies.

Aphids show inconsistent responses to drought stress. Several studies have observed reductions in growth rate or reproductive performance [5,66,67], while others have observed improved performance [68]. Responses are often species-specific. Khan et al. [69] observed that while the specialist *Brevicoryne brassicae* (Hemiptera: Aphididae) was less affected by drought stress, the generalist species *Myzus persicae* (Hemiptera: Aphididae) had larger populations on drought stressed plants. A species-specific response can also be observed in other insect families, such as lepidopterans [70]. Interactions between phytophagous insects can also be altered by drought [71].

The performance of *E. abietinum* in response to drought stress has been assessed over several studies [13,14,15,16]. During a comparable trial, in relation to both the start and timings of the study presented here and in drought treatments used, mean aphid nymph weight was found to be reduced under both continuous and intermittent severe drought stress, while adult aphid weight was slightly, though significantly, increased under the same treatments. Aphid weights of both adults and nymphs was found to significantly increase under moderate intermittent stress.

The consumption rates of both *A. obliterata* and *A. bipunctata* were observed in this study to be significantly higher under the severe stress treatments, for the adults and larvae of both species and regardless of arena substrate. These findings, supported by those in Banfield-Zanin and Leather [14], suggest that under severe drought a greater number of aphids must be consumed in order to meet the dietary requirements of natural enemies. It could also be the case, especially for larvae, that the reduction in aphid size under severe drought may reduce handling time of the prey.

The results observed under moderate intermittent stress are somewhat less clear-cut, although a trend is apparent whereby fewer aphids reared under these conditions were consumed by both adults and larvae. Fewer aphids would need to be consumed as they were larger [14]; however, a statistically significant reduction was only observed in the consumption rates of the larvae, suggesting a potential age-dependent effect. It is likely that, due to their larger size, the aphids were more difficult to handle for the early-instar larvae [72], whereas adults required fewer prey items to become satiated.

The response of plants to drought can be complex. Changes to terpene levels, for example, have been observed in Sitka spruce in response to drought stress. Major [56] observed higher levels under intermittent stress when compared to continuous and control levels of drought stress. Terpenes function as a defensive secondary metabolite, which can also function as volatiles. Herbivore-induced plant volatiles and other semiochemicals are known to affect the behaviour of herbivores and their natural enemies. They can, for example, control host selection for herbivores [73]. They can also enhance the ability of natural enemies to locate their prey on a plant [74]. Changes to the morphology of conifer seedlings has been recorded in response to drought stress [60], although such changes are not necessarily true in all arboreal settings [75]. Trees, through their lifetime, are likely to face a diverse range of conditions and stresses, and, therefore, have means of compensating for these effects.

The consumption rates in empty Petri dishes compared with those with Sitka spruce segments were found to be significantly different. Despite this, the responses followed the same pattern in all cases—more aphids consumed under severe stress, fewer under moderate intermittent stress (even if not significantly so). This would suggest that the differences in consumption rates were driven by differences in the aphids rather than changes to the host plant structure. It is unclear, however, whether drought-induced changes to the chemistry of the Sitka spruce host plants played a role through the aphids.

The host plant segments provided in the Petri dish arenas during the experiments were taken from plants that had undergone a previous year’s worth of drought treatment, in order to reflect any changes to needle morphology. Given that the consumption rates followed the same patterns in both arenas, the implication is that there were no significant differences in the morphology of the segments. At the very least, any changes would not have affected the searching behaviour of the coccinellids.

Functional response studies are often carried out in empty Petri dishes (on the dish surface, e.g., Leather and Owuor [47], Hassell et al. [76]). While this does reduce the number of uncontrolled variables and allows for comparison between previous standardised Petri dish studies, the realised functional response exhibited by the predator in a natural environment may not conform to the observed results. The same holds true in the case of consumption rates. Given that it is the realised response and consumption rate, which, in terms of potential biological control, are the most pertinent, and comparing the two scenarios is important as several factors are altered by the presence of host material. Two such factors are effects of herbivore behaviours and search time.

Phytophagous insect activity differs when in the presence of host plant material in comparison to an empty substrate surface. As an example, *Tetranychus urticae* (Acari: Tetranychidae) mites were found by Everson [77] to be inactive on bean leaves, but active in empty Petri dishes. The same applies in the case of aphids, which are comparatively immobilised during feeding due to the insertion of their stylets into the plant tissue in order to access the phloem [78]. Feeding cannot take place, however, if there is no plant material, and, as such, they may be able to respond to predator disturbance more promptly—in order to escape from attack, a feeding aphid must first remove its stylets from the plant before reacting. While many species walk away, *E. abietinum* exhibits a dropping response to disturbance [79]. Though this may be of benefit in the presence of a Sitka spruce segment, the same does not hold true in an empty Petri dish, where the behaviour would not remove the aphid from the immediate vicinity of the predator.

Beyond the effects on herbivore behaviour, search time for the predator on host plant material must inevitably be increased in comparison with an empty Petri dish. This results from an increased search area, and, in the cases where the host plant is a coniferous species, each needle must be searched individually.

In this experiment, significant differences were observed in aphid consumption rates in all cases dependent on the arena substrate conditions. In the case of adult coccinellids of both species, a greater number of aphids were consumed in the empty Petri dish arenas. The difference was less pronounced for *A. obliterata* adults, with only a difference of 8.3% in the mean consumption rates of the two substrate types. *Adalia bipunctata*, on the other hand, nearly doubled the difference, consuming 18.5% more aphids in an empty Petri dish. The responses of the 1st instar larvae, on the other hand, were species-dependent. Considerably more aphids, 25.5% more, were eaten by *A. bipunctata* larvae. In contrast to this, and to what was observed with the adults, *A. obliterata* larvae consumed 4.6% fewer aphids in an empty Petri dish. Not only that, but *A. obliterata* larvae consumed a greater number of aphids in both cases than *A. bipunctata* did. It is possible that differences between the specialist *A. obliterata* and the generalist *A. bipunctata* can, in part, explain these differences. Prey handling time should not have been greatly affected in the case of the adult coccinellids, but prey searching time would have been reduced in an empty Petri dish. In the presence of Sitka spruce plant material, the specialist predator may well have prey-searching behaviour better adapted to searching for the aphids amongst the spruce needles in comparison to the generalist predator. This may be more strongly demonstrated in the case of the larval coccinellids, whereby the small *A. obliterata* larvae nonetheless consumed greater numbers of aphids than the larger *A. bipunctata* larvae. The former did, however, show a drop in consumption when host plant material was not present, which further supports an influence of prey-searching behavioural differences. The findings also suggest that, under field conditions, mortality may be higher for early instar *A. bipunctata*, thus decreasing the species’ pest control potential as lower consumption rates are associated with increased mortality [80], positively impacting the fitness of *A. obliterata*, in turn [28].

The observed results of this study suggest that *E. abietinum* were able to capitalise on the presence of host plant material in all cases except when preyed upon by 1st instar *A. obliterata* larvae, with host plant presence either potentially facilitating an escape response or increasing the prey search time for coccinellids. Furthermore, *A. bipunctata* were less able to respond to the presence of plant material (with a larger difference between the number of aphids consumed in the presence or absence of host plant material when compared to *A. obliterata*), and although adults of this species consumed a greater number of aphids than the spruce specialist, their larval counterparts performed worse than the *A. obliterata* larvae.

It should be noted that these findings reflect a trophic response under controlled laboratory conditions, and thus ecological significance or responses under uncontrolled, field conditions are likely to show a degree of variance from the findings herein presented. This would likely be partially attributable to interactive effects of climatic variables—for example, the combined effects of drought, temperature and atmospheric gas levels, which would alter life history traits for all trophic levels simultaneously. The natural inclusion of additional trophic levels would moderate the presented plant–herbivore–predator relationship through altered phenological and physiological timings [81,82]. Furthermore, the physiological responses to altered variables may differ between trophic levels to the same climatic alterations, raising inconsistencies across scenarios and timescales [83,84]. The importance of such interactive effects has been noted in various studies and meta-analyses, with some indicating synergistic effects [85], while a growing body of evidence suggests antagonistic effects [86,87].

## 5. Conclusions

Drought, as predicted under climate change, is likely to alter the prey consumption in Sitka spruce plantations. Severe levels of drought stress, both continuous and intermittent, resulted in an increase in the consumption of *E. abietinum* by both *A. obliterata* and *A. bipunctata* under controlled conditions, whereas a (non-statistically significant) trend for reduced consumption of prey was observed under moderate intermittent drought stress.

## Figures and Tables

**Table 1:** Mean consumption rates by adult coccinellids of *E. abietinum* raised under drought stress in 24 h. FC = field capacity; MS = 60% field capacity; CS = 20% field capacity; MIS = allowed to fluctuate from 70% to 30% field capacity; SIS = allowed to fluctuate from field capacity to 20% field capacity.

	*A. obliterata*		*A. bipunctata*	
Drought	with Host Plant	without Host Plant	with Host Plant	without Host Plant
	$\bar{x}$ ± SE	$\bar{x}$ ± SE	$\bar{x}$ ± SE	$\bar{x}$ ± SE
FC	26.67 ± 1.01	29.75 ± 0.91	45.90 ± 1.23	54.01 ± 0.97
MS	30.21 ± 1.15	32.76 ± 1.08	47.64 ± 1.26	58.58 ± 0.85
CS	35.43 ± 1.20	36.62 ± 0.95	54.02 ± 1.18	63.66 ± 1.14
MIS	24.02 ± 1.26	30.51 ± 1.15	46.02 ± 1.08	53.32 ± 1.04
SIS	34.54 ± 1.35	35.66 ± 1.19	50.77 ± 1.31	60.26 ± 1.36

**Table 2:** Mean consumption rates by 1st instar coccinellid larvae of *E. abietinum* raised under drought stress in 24 h. FC = field capacity; MS = 60% field capacity; CS = 20% field capacity; MIS = allowed to fluctuate from 70% to 30% field capacity; SIS = allowed to fluctuate from field capacity to 20% field capacity.

Drought	*A. obliterata*		*A. bipunctata*	
	with Host Plant	without Host Plant	with Host Plant	without Host Plant
	$\bar{x}$ ± SE	$\bar{x}$ ± SE	$\bar{x}$ ± SE	$\bar{x}$ ± SE
FC	10.46 ± 0.22	9.58 ± 0.19	5.34 ± 0.18	7.74 ± 0.21
MS	10.15 ± 0.18	10.57 ± 0.21	5.23 ± 0.20	7.68 ± 0.26
CS	11.83 ± 0.25	10.66 ± 0.24	7.09 ± 0.26	9.31 ± 0.18
MIS	8.94 ± 0.14	8.48 ± 0.23	4.88 ± 0.14	6.19 ± 0.25
SIS	10.80 ± 0.28	10.48 ± 0.20	6.66 ± 0.22	8.46 ± 0.21
